# SORTA: a system for ontology-based re-coding and technical annotation of biomedical phenotype data

**DOI:** 10.1093/database/bav089

**Published:** 2015-09-17

**Authors:** Chao Pang, Annet Sollie, Anna Sijtsma, Dennis Hendriksen, Bart Charbon, Mark de Haan, Tommy de Boer, Fleur Kelpin, Jonathan Jetten, Joeri K. van der Velde, Nynke Smidt, Rolf Sijmons, Hans Hillege, Morris A. Swertz

**Affiliations:** ^1^University of Groningen, University Medical Centre Groningen, Genomics Coordination Centre, Department of Genetics, Groningen, The Netherlands,; ^2^University of Groningen, University Medical Centre Groningen, Department of Genetics, Groningen, The Netherlands,; ^3^University of Groningen, University Medical Center Groningen, Department of Epidemiology, Groningen, The Netherlands and; ^4^LifeLines Cohort Study and Biobank, Groningen, The Netherlands

## Abstract

There is an urgent need to standardize the semantics of biomedical data values, such as phenotypes, to enable comparative and integrative analyses. However, it is unlikely that all studies will use the same data collection protocols. As a result, retrospective standardization is often required, which involves matching of original (unstructured or locally coded) data to widely used coding or ontology systems such as SNOMED CT (clinical terms), ICD-10 (International Classification of Disease) and HPO (Human Phenotype Ontology). This data curation process is usually a time-consuming process performed by a human expert. To help mechanize this process, we have developed SORTA, a computer-aided system for rapidly encoding free text or locally coded values to a formal coding system or ontology. SORTA matches original data values (uploaded in semicolon delimited format) to a target coding system (uploaded in Excel spreadsheet, OWL ontology web language or OBO open biomedical ontologies format). It then semi- automatically shortlists candidate codes for each data value using Lucene and n-gram based matching algorithms, and can also learn from matches chosen by human experts. We evaluated SORTA’s applicability in two use cases. For the LifeLines biobank, we used SORTA to recode 90 000 free text values (including 5211 unique values) about physical exercise to MET (Metabolic Equivalent of Task) codes. For the CINEAS clinical symptom coding system, we used SORTA to map to HPO, enriching HPO when necessary (315 terms matched so far). Out of the shortlists at rank 1, we found a precision/recall of 0.97/0.98 in LifeLines and of 0.58/0.45 in CINEAS. More importantly, users found the tool both a major time saver and a quality improvement because SORTA reduced the chances of human mistakes. Thus, SORTA can dramatically ease data (re)coding tasks and we believe it will prove useful for many more projects.

**Database URL**: http://molgenis.org/sorta or as an open source download from http://www.molgenis.org/wiki/SORTA

## Introduction

Biobank and translational research can benefit from the massive amounts of phenotype data now being collected by hospitals and via questionnaires. However, heterogeneity between datasets remains a barrier to integrated analysis. For the BioSHaRE (1) biobank data integration project, we previously developed BiobankConnect (2), a tool to overcome heterogeneity in data *structure* by mapping data *elements* from the source database onto a target scheme. Here, we address the need to overcome heterogeneity of data *contents* by coding and/or recoding data *values*, i.e. mapping free text descriptions or locally coded data values onto a widely used coding system. In this ‘knowledge-based data access’, data is collected and stored according to local requirements while information extracted from the data is revealed using standard representations, such as ontologies, to provide a unified view (3).

The (re)coding process is essential for the performance of three different kinds of functions:

- *Search and query.* The data collected in a research and/or clinical setting can be described in numerous ways with the same concept often associated with multiple synonyms, making it difficult to query distributed database systems in a federated fashion. For example, using standard terminologies, the occurrence of ‘cancer’ written in different languages can be easily mapped between databases if they have been annotated with same ontology term.
- *Reasoning with data*. Ontologies are the formal representation of knowledge and all of the concepts in an ontology have been related to each other using different relationships, e.g. ‘A is a *subclass of* B’. Based on these relationships, the computer can be programmed to reason and infer the knowledge (4). For example, when querying cancer patients’ records from hospitals, those annotated with ‘Melanoma’ will be retrieved because ‘Melanoma’ is specifically defined as a descendant of ‘Cancer’ in the ontology.
- *Exchange or pooling of data across systems*. Ontologies can also be used to describe the information model, such as the MGED (Microarray Gene Expression Data) ontology describing microarray experiments or hospital information coded using the ICD-10 (International Classification of Diseases) coding system, so that the data can easily flow across systems that use the same model (4).

The data (re)coding task is essentially a matching problem between a list of free text data values to a coding system, or from one coding system to another. Unfortunately, as far as we know, there are only a few software tools available that can assist in this (re)coding process. Researchers still mostly have to evaluate and recode each data value by hand, matching values to concepts from the terminology to find the most suitable candidates. Not surprisingly, this is a time-consuming and error-prone task. Based on our previous success in BioSHaRE, we were inspired to approach this problem using ontology matching and lexical matching (2). We evaluated how these techniques can aid and speed-up the (re)coding process in the context of phenotypic data. In particular, we used our newly developed system, SORTA, to recode 5210 unique entries for ‘physical exercise’ in the LifeLines biobank (5) and 315 unique entries for ‘physical symptoms’ (including terms that are similar, but not the same) in the Dutch CINEAS (www.cineas.org) (6) and HPO (Human Phenotype Ontology) coding systems for metabolic diseases.

### Requirements

Several iterations of SORTA-user interviews resulted in the identification of the following user requirements:

1. Comparable similarity scores, e.g. scores expressed as a percentage, so users can easily assess how close a suggested match is to their data, and decide on a cut-off to automatically accept matches.
2. Support import of commonly used ontology formats (OWL/OBO) for specialists and Excel spread sheets for less technical users.
3. Fast matching algorithm to accommodate large input datasets and coding systems.
4. Online availability so users can recode/code data directly and share with colleagues without need to download/install the tool.
5. Maximize the sensitivity to find candidate matches and let users decide on which one of them is the ‘best’ match.
6. Enable complex matching in which not only a text string is provided but also associated attributes such as labels, synonyms and annotations, e.g. [label: Hearing impairment, synonyms:(Deafness, Hearing defect)].

### Approaches

Two types of matching approaches have been reported in the literature: lexical matching and semantic matching. *Lexical matching* is a process that measures the similarity between two strings (7). Edit-distance (8), n-gram (9) and Levenshtein distance (10) are examples of string-based algorithms that focus on string constituents and are often useful for short strings, but they do not scale up for matching large numbers of entity pairs. Token-based techniques focus on word constituents by treating each string as a bag of words. An example of these techniques is the vector space model algorithm (11), in which each word is represented as a dimension in space and a cosine function is used to calculate the similarity between two string vectors. Lexical matching is usually implemented in combination with a normalization procedure such as lowering case, removing stop words (e.g. ‘and’, ‘or’, ‘the’) and defining word stems (e.g. ‘smoking’ → ‘smoke’). *Semantic matching* techniques search for correspondences based not only on the textual information associated to a concept (e.g. description) but also on the associative relationships between concepts (e.g. subclass, ‘is-a’) (7). In these techniques, for example, ‘melanoma’ is a good partial match for the concept called ‘cancer’. Because our goal is to find the most likely concepts matching data values based on their similarity in description, lexical-based approaches seem most suitable.

One of the challenges in the (re)coding task is the vast number of data values that need to be compared, which means that the matcher has to find correspondences between the Cartesian product of the original data values and the codes in the desired coding system. High-throughput algorithms are needed to address this challenge and two methods have been developed to deal with the matching problem on a large scale. The Early Pruning Matching Technique (12) reduces search space by omitting irrelevant concepts from the matching process, e.g. the ontology concept (label:hearing impairment, synonyms[deafness, hearing defect, congenital hearing loss]) that does not contain any words from the search query ‘protruding eye ball’ are eliminated. The Parallel Matching Technique (12) divides the whole matching task into small jobs and the matcher then runs them in parallel, e.g. 100 data values are divided into 10 partitions that are matched in parallel with ontologies.

### Existing tools

We found several existing tools that offered partial solutions, see Table 1. Mathur and Joshi (13) described an ontology matcher, Shiva, that incorporates four string-matching algorithms (Levenshtein distance, Q-grams, Smith Waterman and Jaccard), any of which could be selected by users for particular matching tasks. They used general resources like WordNet and Online Dictionary to expand the semantics of the entities being matched. Cruz (14) described a matcher, Agreement Maker, in which lexical and semantic matchers were applied to ontologies in a sequential order and the results were combined to obtain the final matches. At the lexical matching stage, Cruz (14) applied several different kinds of matchers, string-based matches (e.g. edit distance and Jar-Winkler) and an internally revised token-based matcher, then combined the similarity metrics from these multiple matchers. Moreover the philosophy behind this tool is that users can help make better matches in a semi-automatic fashion that are not possible in automatic matching (14). Jiménez-Ruiz and Cuenca Gra (15) described an approach where: (i) they used lexical matching to compute an initial set of matches; (ii) based on these initial matches, they took advantage of semantic reasoning methods to discover more matches in the class hierarchy and (iii) they used indexing technology to increase the efficiency of computing the match correspondences between ontologies. Peregrine (16) is an indexing engine or tagger that recognizes concepts within human readable text, and if terms match multiple concepts it tries to disambiguate BioPortal (17), the leading search portal for ontologies, provides the BioPortal Annotator that allows users to annotate a list of terms with pre-selected ontologies. While it was useful for our use cases, it was limited because it only retrieves perfect matches and terms with slightly different spellings cannot be easily matched (e.g. ‘hearing impaired’ vs. ‘hearing impairment’) (18). In addition, BioPortal Annotator’s 500-word limit reduces its practical use when annotating thousands of data values. Finally, ZOOMA (19) enables semi-automatic annotation of biological data with selected ontologies and was closest to our needs. ZOOMA classifies matches as ‘Automatic’ or ‘Curation required’ based on whether or not there is manually curated knowledge that supports the suggested matches. ZOOMA does not meet our requirements in that it does not provide similarity scores for the matches, does not prioritize recall over precision (i.e. ZOOMA matches are too strict for our needs), and does not handle partial/complex matches. For example, in ZOOMA, the OMIM (Online Mendelian Inheritance in Man) term ‘Angular Cheilitis’ could not be partially matched to the HPO term ‘Cheilitis’ and ‘Extra-Adrenal Pheochromocytoma’ could not be matched to the HPO term ‘Extraadrenal pheochromocytoma’ because of the hyphen character.

**Table 1.** Comparison of existing tools with SORTA

	SORTA	BioPortal annotator	ZOOMA	Shiva	Agreement maker	LogMap	Peregrine
Comparable similarity score	Y	N	N	N	Y	Y	N
Import code system in ontology format	Y	Y	Y	Y	Y	Y	Y
Import code system in excel format	Y	N	N	N	N	N	N
Uses lexical index to improve performance	Y	Y	Y	N	N	Y	Y
Code/Recode data directly in the tool	Y	N	N	N	Y	N	N
Tool available as online service	Y	Y	Y	N/A	N/A	N/A	N
Support partial matches	Y	N	N	Y	Y	Y	N
Match complex data values	Y	N	N	Y	Y	Y	N
Learns from curated dataset	Y	N	Y	N	N	N	N

Y represents Yes; N represents No; N/A represents unknown

ZOOMA and BioPortal Annotator were the closest to our needs.

## Method

Based on our evaluation of existing tools, we decided to combine a token-based algorithm, Lucene (20), with an n-gram-based algorithm. Lucene is a high-performance search engine that works similarly to the Early Pruning Matching Technique. Lucene only retrieves concepts relevant to the query, which greatly improves the speed of matching. This enables us to only recall suitable codes for each value and sort them based on their match. However, the Lucene matching scores are not comparable across different queries making it unsuitable for human evaluation. Therefore, we added an n-gram-based algorithm as a second matcher, which allows us to standardize the similarity scores as percentages (0–100%) to help users understand the quality of the match and to enable a uniform cut-off value.

**Figure 1.**
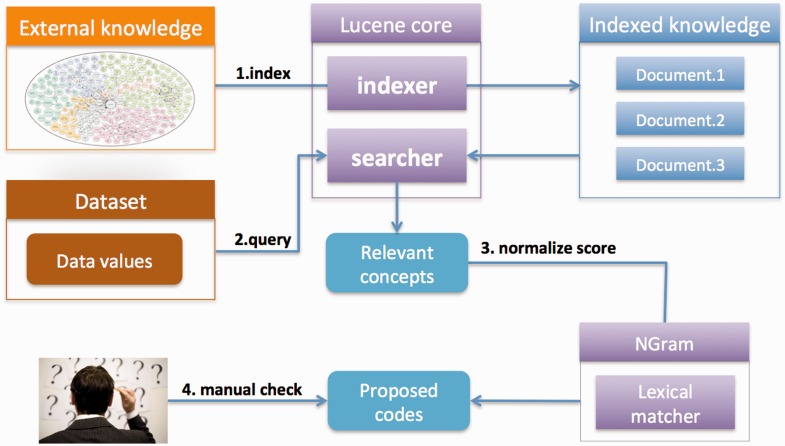
SORTA overview. The desired coding system or ontology can be uploaded in OWL/OBO and Excel and indexed for fast matching searches. Data values can be uploaded and then automatically matched with the indexed ontology using Lucene. A list of the most relevant concepts is retrieved from the index and matching percentages are calculated using the n-gram algorithm so that users can easily evaluate the matching score. Users can choose the mappings from the suggested list.

We implemented the following three steps. First, coding systems or ontologies are uploaded and indexed in Lucene to enable fast searches (once for each ontology). Second, users create their own coding/recoding project by uploading a list of data values. What users get back is a shortlist of matching concepts for each value that has been retrieved from the selected coding system based on their lexical relevance. In addition, the concepts retrieved are matched with the same data values using the second matcher, the n-gram-based algorithm, to normalize the similarity scores to values from 0 to 100%. Finally, users apply a %-similarity-cut-off to automatically accept matches and/or manually curates the remaining codes that are assigned to the source values. Finally, users download the result for use in their own research. An overview of the strategy is shown in Figure 1. We provide a detailed summary below.

Users upload coding sources such as ontologies or terminology lists to establish the knowledge base. Ontologies are the most frequently used source for matching data values, but some of the standard terminology systems are not yet available in ontology formats. Therefore, we allow users to not only upload ontologies in OWL and OBO formats, but also import a ‘raw knowledge base’ stored in a simple Excel format which includes system ID, concept ID and label (see Table 2). The uploaded data is then indexed and stored locally to enable rapid matching.

**Table 2.** Example of how to upload a coding system and a coding/recoding target

Concept ID	Concept Label	System ID
02060	cardio training	MET
02020	bodypump	MET
18310	swimming	MET
15430	kung fu	MET
15350	hockey	MET
12150	running	MET

This example shows an Excel file with MET (Metabolic Equivalent of Task), a system developed to standardize physical activity, in which each concept ID includes a list of different sports representing specific amounts of energy consumption.

To match data values efficiently, we used the Lucene search index with the default snowball stemmer and a standard filter for stemming and removing stop words. A code/ontology concept is evaluated as being a relevant match for the data value when it or its corresponding synonyms (if available) contain at least one word from the data value. The assumption in this strategy is that the more words a concept’s label or synonyms contain, the more relevant Lucene will rank it, and therefore the top concepts on the list are most likely to be the correct match. However, the snowball stemmer could not stem some of the English words properly, e.g. the stemmed results for ‘placenta’ and ‘placental’ were ‘placenta’ and ‘placent’, respectively. To solve this problem, we enabled fuzzy matching with 80% similarity and this allowed us to maximize the number of relevant concepts retrieved by Lucene.

Lucene also provides matching scores that are calculated using a cosine similarity between two weighted vectors (21), which takes the information content of words into account, e.g. rarer words are weighted more than common ones. However, after our first user evaluations we decided not to show Lucene scores to users for two reasons. First, Lucene calculates similarity scores for any indexed document as long as it contains at least one word from the query. Documents that have more words that match the query, or contain words that are relatively rare, will get a higher score. Second, the matching results produced by different queries are not comparable because the scales are different (22) making it impossible to determine the ‘best’ cut-off value above which the suggested matches can be assumed to be correct.

We therefore decided to provide an additional similarity score that ranges from 0 to 100% by using an n-gram calculation between the data value and the relevant concepts retrieved by Lucene. In this n-gram-based algorithm, the similarity score is calculated for two strings each time. The input string is lowercased and split by whitespace to create a list of words, which are then stemmed by the default snowball stemmer. For each of the stemmed words, it is appended with ‘^’ at the beginning and ‘$’ at the end, from which the bigram tokens are generated, e.g. ^smoke$ → [^s, sm, mo, ok, ke, e$]. All the bigram tokens are pushed to a list for the corresponding input string with duplicated tokens allowed. The idea is that the more similar two strings are, the more bigram tokens they can share. The similarity score is the product of number of shared bigram tokens divided by the sum of total number of bigram tokens of two input strings as follows,

$$\text{Similarity}=\;\frac{\text{Number}\;\text{of}\;\text{shared}\;\text{bigram}\;\text{tokens}\times 2}{\begin{array}{l} \text{Number}\;\text{of}\;\text{bigram}\;{\text{tokens}}_{S1}+\\ \text{Number}\;\text{of}\;\text{bigram}\;{\text{tokens}}_{S2} \end{array}}$$

Because we were only interested in the constituents of the strings being compared, the order of the words in strings does not change the score. We also considered only using the n-gram calculation, but that would require calculation of all possible pairwise comparisons between all data values and codes, which would greatly slow down the process.

Ultimately both algorithms were combined because Lucene is very efficient in retrieving relevant matches while our users preferred n-gram scores because they are easier to compare. Combining Lucene with the n-gram-based algorithm is an optimal solution in which the advantages of both methods complement each other while efficiency, accuracy and comparability of scores are preserved.

To code the data values, the data can be uploaded as a simple comma separate value file or copy/pasted into the text area directly in SORTA. The uploaded data is usually a list of simple string values, however in some cases it also can be complex data values containing information other than a simple label.

For these cases, SORTA allows inclusion of descriptive information such as synonyms and external database identifiers to improve the quality of the matched results shown in Table 3.

**Table 3.** Example of how to upload data values and coding/recoding source)

Name (required)	Synonym_1 (optional)	OMIM (optional)
2,4-dienoyl-CoA reductase deficiency	DER deficiency	222745
3-methylcrotonyl-CoA carboxylase deficiency	3MCC	210200
Acid sphingomyelinase deficiency	ASM	607608

At minimum, one column of values should be provided: the first column with the header ‘Name’. Additional optional columns that start with ‘Synonym_’ can contain the synonyms for input values. Other optional column headers can contain other identifiers, e.g. in this example OMIM.

For each of the data values, a suggested list of matching concepts is retrieved and sorted based on similarity. Users can then check the list from the top downwards and decide which of the concepts should be selected as the final match. However, if the first concept on the list is associated with a high similarity score, users can also choose not to look at the list because they can confidently assume that a good match has been found for that data value. By default, 90% similarity is the cut-off above which the first concept on the retrieved list is automatically picked as the match for the data value and stored in the system. Below 90% similarity, users are required to manually check the list to choose the final match. The cut-off value can be changed according to the needs of the project, e.g. a low cut-off of 70% can be used if the data value was collected using free text because typos are inevitably introduced during data collection.

## Results

We evaluated SORTA in various projects. Here we report two representative matching scenarios where the original data values were either free text (case 1) or already coded, but using a local coding system (case 2). In addition, as a benchmark, we generated matches between HPO, NCIT (National Cancer Institute Thesaurus), OMIM (Online Mendelian Inheritance in Man) and DO (Disease Ontology) and compared the matches with existing cross references between these two (case 3)

## Case 1: Coding unstructured data in the LifeLines biobank

### Background

LifeLines is a large biobank and cohort study started by the University Medical Centre Groningen, the Netherlands. Since 2006, it has recruited 167 729 participants from the northern region of the Netherlands (5). LifeLines is involved in the EU BioSHaRE consortium and one of the joint data analyses being conducted by BioSHaRE is the ‘Healthy Obese Project’ (HOP) that examines why some obviously obese individuals are still metabolically healthy (23). One of the variables needed for the HOP analysis is physical activity but, unfortunately, this information was collected using a Dutch questionnaire containing free text fields for types of sports. Researchers thus needed to match these to an existing coding system: the Ainsworth compendium of physical activities (24). In this compendium each code matches a metabolic equivalent task (MET) intensity level corresponding to the energy cost of that physical activity and defined as the ratio of the metabolic rate for performing that activity to the resting metabolic rate. One MET is equal to the metabolic rate when a person is quietly sitting and can be equivalently expressed as:

$$1\text{MET}\equiv 1\frac{\text{kcal}}{\text{kg}\times \text{h}}\equiv 4.184\;\frac{\text{kJ}}{\text{kg}\times \text{h}}$$

A list of 800 codes has been created to represent all kinds of daily activities with their corresponding energy consumption (24). Code 1015, for example, represents ‘general bicycling’ with a MET value of 7.5. The process of matching the physical activities of LifeLines data with codes is referred to as coding.

### Challenges and motivation

There were two challenges in this task. First, the physical activities were collected in Dutch and therefore only researchers with a good level of Dutch could perform the coding task. Second, there were data for more than 90 000 participants and each participant could report up to four data values related to ‘Sport’ that could be used to calculate the MET value. In total, there were 80 708 terms (including 5211 unique terms) that needed to be coded. We consulted with the researchers and learned that they typically coded data by hand in an Excel sheet or by syntax in SPSS, and for each entry they needed to cross-check the coding table and look up the proper code. While this approach is feasible on a small scale (<10 000 participants), it became clear it would be too much work to manually code such a massive amount of data. Hence, we used our SORTA coding system.

To train SORTA, we reused a list of human-curated matches between physical activities described in Dutch and the codes that were created for a previous project. We used this as the basis to semi-automatically match the new data from LifeLines. An example of the curated matches is shown in Table 2 and the complete list can be found at Supplementary material: Lifelines_MET_mappings.xlsx. Moreover, we have enhanced SORTA with an upload function to support multiple ‘Sport’-related columns in one harmonization project. This can be done as long as the column headers comply with the standard naming scheme, where the first column header is ‘Identifier’ and other column headers start with string ‘Sport_’, e.g. ‘Sport_1’ and ‘Sport_2’.

**Figure 2.**
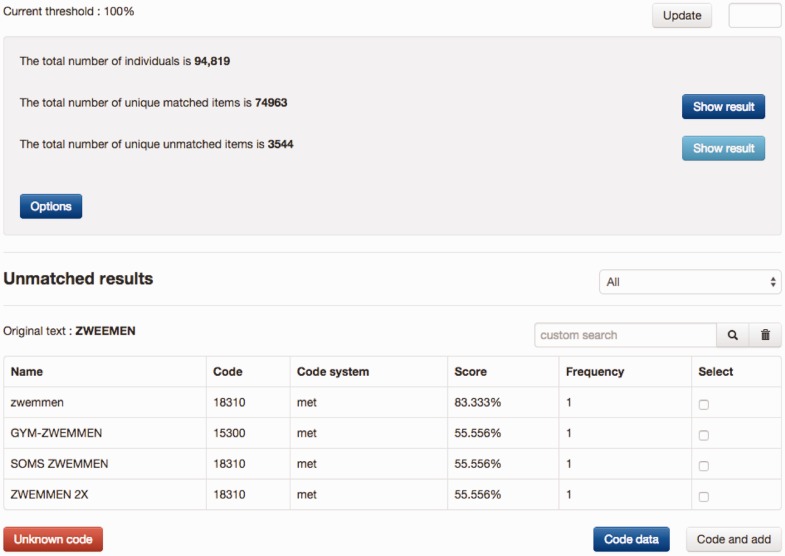
Example of coding a physical activity. A list of MET codes was matched with input and sorted based on similarity scores, from which the proper code can be selected to recode the input. If none of the candidate codes is suitable, users can either search for codes manually or decide to use ‘Unknown code’. If the button ‘Code data’ is clicked, the input is recoded only with the selected code. If the button ‘Code and add’ is clicked, the input is recoded and the input gets added to the code as a new synonym. The example is a typo of the Dutch word for ‘swimming’. zwemmen = swimming, zwemmen 2x = twice a week, soms zwemmen = occasional swimming, gym-zwemmen = water gym.

Figure 2 shows an example of manually coding the physical activity ‘ZWEMMEN’ (Swimming) with MET codes, in which a shortlist of candidates were retrieved by SORTA and the first item of the list selected as the true match. Each time the manual curation process produced a new match, this new knowledge could be added to the knowledge base to be applied to all future data values. This is an optional action because data values (especially those filled in by participants of the study) sometimes contain spelling errors that should not be added to the knowledge base.

### Evaluation

With the assistance of SORTA, all of the data values have been coded by the researcher who is responsible for releasing data about physical activity in the LifeLines project. The coding result containing a list of matches was used as the gold standard for the following analysis, in which we evaluated two main questions: (i) How far could the previous coding round improve the new matching results? (ii) What is the best cut-off value above which the codes selected by SORTA can be confidently assumed to be correct matches to a value?

SORTA’s goal is to shortlist good codes for the data values so we first evaluated the rank of the correct manual matches because the higher they rank, the less manual work the users need to perform. Our user evaluations suggested that as long as the correct matches were captured in the top 10 codes, the researchers considered the tool useful. Otherwise, based on their experience, users changed the query in the tool to update the matching results.

**Figure 3.**
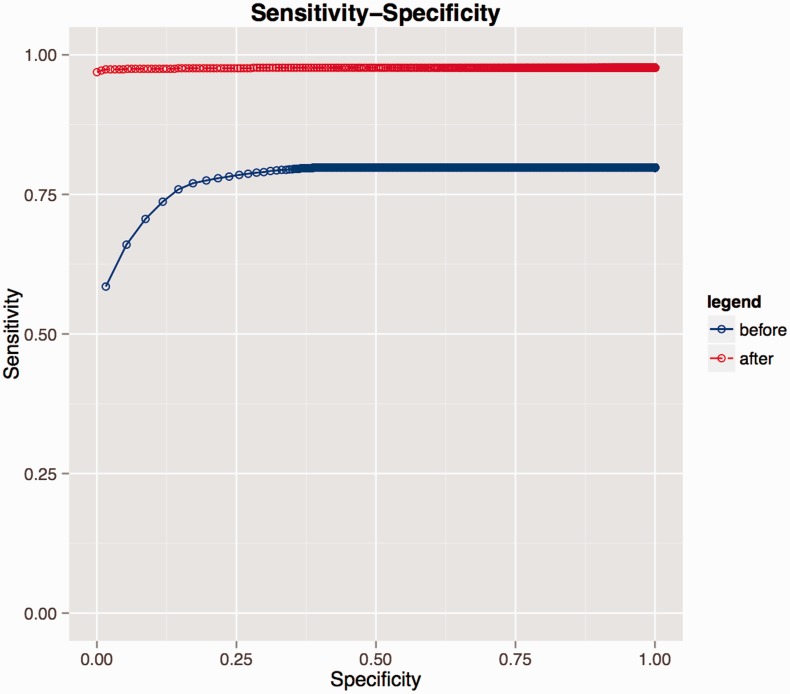
Receiver operating characteristic (ROC) curves evaluating performance on LifeLines data. Blue represents the performance before the researcher recoded all the LifeLines data. During coding, the researcher introduced new knowledge to the database and if a similar dataset was uploaded again (e.g. second rounds of the same questionnaire), the coding performance greatly improved as shown by the red curve.

Re-use of manually curated data from the previous coding round resulted in an improvement in SORTA’s performance with recall/precision at rank 1^st^ increasing from 0.59/0.65 to 0.97/0.98 and at rank 10th from 0.79/0.14 to 0.98/0.11 (see Figure 3 and Table 4). At the end of the coding task, about 97% of correct matches were captured at rank 1st with users only needing to look at the first candidate match.

**Table 4.** Precision and recall for the LifeLines case study

Rank cut-off	Before coding			After coding		
	Recall	Precision	F-measure	Recall	Precision	F-measure
1	0.59	0.65	0.62	0.97	0.98	0.97
2	0.66	0.39	0.49	0.97	0.50	0.66
3	0.71	0.29	0.41	0.97	0.34	0.50
4	0.74	0.24	0.36	0.97	0.26	0.41
5	0.76	0.21	0.33	0.97	0.21	0.35
6	0.77	0.19	0.30	0.97	0.18	0.30
7	0.78	0.17	0.28	0.97	0.15	0.26
8	0.78	0.16	0.27	0.98	0.14	0.25
9	0.78	0.14	0.24	0.98	0.12	0.21
10	0.79	0.14	0.24	0.98	0.11	0.20
11	0.79	0.13	0.22	0.98	0.10	0.18
12	0.79	0.12	0.21	0.98	0.09	0.16
13	0.79	0.12	0.21	0.98	0.09	0.16
14	0.79	0.12	0.21	0.98	0.08	0.15
15	0.79	0.11	0.19	0.98	0.08	0.15
16	0.79	0.11	0.19	0.98	0.07	0.13
17	0.79	0.11	0.19	0.98	0.07	0.13
18	0.80	0.11	0.19	0.98	0.06	0.11
19	0.80	0.10	0.18	0.98	0.06	0.11
20	0.80	0.10	0.18	0.98	0.06	0.11
30	0.80	0.10	0.18	0.98	0.04	0.08
50	0.80	0.09	0.16	0.98	0.03	0.06

In total, 90 000 free text values (of which 5211 were unique) were recoded to physical exercise using MET coding system. The table shows recall and precision per position in the SORTA result before coding (using only the MET score descriptions) and after coding (when a human curator had already processed a large set of SORTA recommendations by hand).

We included use of an n-gram-based algorithm to provide users with an easily understood metric with which to judge the relevance of the proposed codes on a scale of 1–100%, based on the n-gram match between value and code (or a synonym thereof). Supplementary Table S1 suggests that, in the LifeLines case, 82% similarity is a good cut-off for automatically accepting the recommended code because 100% of the matches produced by the system were judged by the human curator to be correct matches. Because LifeLines data is constantly being updated (with new participants, and with new questionnaire data from existing participants every 18 months), it would be really helpful to recalibrate the cut-off value when the tool is applied anew.

## Case 2: Recoding from CINEAS coding system to HPO ontology

### Background

CINEAS is the Dutch centre for disease code development and its distribution to the clinical genetics community (www.cineas.org) (6). This centre was initiated by the eight clinical genetics centres responsible for genetic counselling and diagnostics in the Netherlands in 1992 (25). CINEAS codes are used in daily practice by Dutch clinical geneticists and genetic counsellors to assign diseases and clinical symptoms to patients. The 63^rd^ edition of CINEAS now lists more than 5600 diseases and more than 2800 clinical symptoms. The challenge was to match and integrate (or recode) the CINEAS clinical symptom list with HPO in order to use one enriched standardized coding system for future coding of patients’ symptoms and to obtain interoperability for CINEAS codes already registered in local systems all over the country. The metabolic diseases obtained from CINEAS disease list, which has become an independent project called The Dutch Diagnosis Registration Metabolic Diseases (DDRMD, https://ddrmd.nl/) (25), will be matched with Orphanet ontology in the future.

### Challenge and motivation

**Figure 4.**
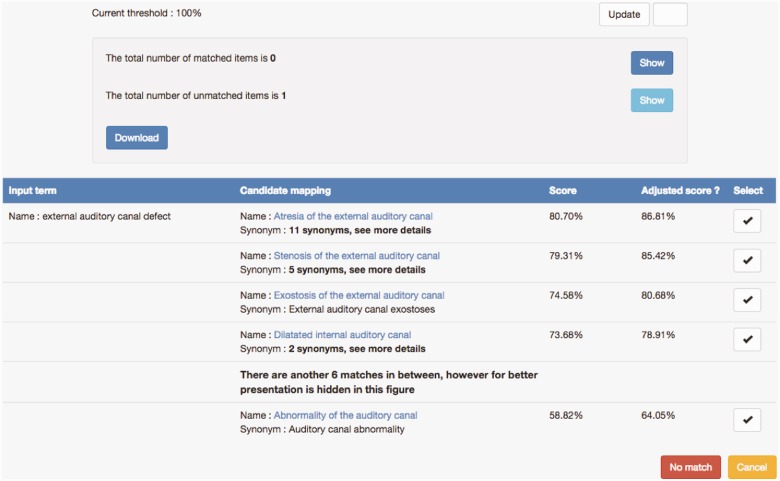
Example of matching the input value ‘external auditory canal defect’ with HPO ontology terms. A list of candidate HPO ontology terms was retrieved from the index and sorted based on similarity scores. Users can select a mapping by clicking the ‘v’ button. If none of the candidate mappings are suitable, users can choose the ‘No match’ option.

The previous strategy of CINEAS curators was to search HPO via BioPortal, however, tracking possible candidate terms meant making written notes or keeping a digital registry on the side, tracking methods that are time- consuming, prone to human errors and demand a lot of switching between tools or screens. Therefore, SORTA was brought into the project. Figure 4 shows an example of a data value ‘external auditory canal defect’ and a list of HPO ontology terms as candidate matches. While none of them is a perfect match for the input term, the top three candidates are the closest matches, but are too specific for the input. Scrutiny by experts revealed that ‘Abnormality of auditory canal’ could be a good ‘partial’ match because of its generality.

### Evaluation

In an evaluation study, the first 315 clinical symptoms out of 2800 were re-coded by a human expert, in which 246 were matched with HPO terms while 69 could not be matched. In addition, we performed the same matching task using BioPortal Annotator and ZOOMA because these existing tools seemed most promising (see Table 5). We further investigated which cut-off value can be confidently used to assume that the automatic matches are correct by calculating precision and recall for all possible n-gram cut-offs (0–100%). Supplementary Table S2 shows 89% to be a good cut-off value for future CINEAS matching tasks because above this value all of the suggested matches are correct with 100% precision.

**Table 5.** Comparison of SORTA, BioPortal and ZOOMA

Rank cut-off	SORTA			BioPortal			ZOOMA		
	Recall	Precision	F-measure	Recall	Precision	F-measure	Recall	Precision	F-measure
1	0.58	0.45	0.51	0.34	0.54	0.42	0.17	0.63	0.27
2	0.69	0.27	0.39	0.35	0.44	0.39	0.17	0.60	0.26
3	0.73	0.19	0.30	0.35	0.44	0.39	0.18	0.60	0.28
4	0.76	0.15	0.25	N/A	N/A	N/A	N/A	N/A	N/A
5	0.78	0.13	0.22	N/A	N/A	N/A	N/A	N/A	N/A
6	0.81	0.11	0.19	N/A	N/A	N/A	N/A	N/A	N/A
7	0.81	0.09	0.16	N/A	N/A	N/A	N/A	N/A	N/A
8	0.83	0.08	0.15	N/A	N/A	N/A	N/A	N/A	N/A
9	0.83	0.08	0.15	N/A	N/A	N/A	N/A	N/A	N/A
10	0.85	0.07	0.13	N/A	N/A	N/A	N/A	N/A	N/A
11	0.85	0.06	0.11	N/A	N/A	N/A	N/A	N/A	N/A
12	0.85	0.06	0.11	N/A	N/A	N/A	N/A	N/A	N/A
13	0.86	0.06	0.11	N/A	N/A	N/A	N/A	N/A	N/A
14	0.86	0.05	0.09	N/A	N/A	N/A	N/A	N/A	N/A
15	0.87	0.05	0.09	N/A	N/A	N/A	N/A	N/A	N/A
16	0.87	0.05	0.09	N/A	N/A	N/A	N/A	N/A	N/A
17	0.87	0.05	0.09	N/A	N/A	N/A	N/A	N/A	N/A
18	0.88	0.04	0.08	N/A	N/A	N/A	N/A	N/A	N/A
19	0.88	0.04	0.08	N/A	N/A	N/A	N/A	N/A	N/A
20	0.88	0.04	0.08	N/A	N/A	N/A	N/A	N/A	N/A
30	0.89	0.03	0.06	N/A	N/A	N/A	N/A	N/A	N/A
50	0.92	0.02	0.04	N/A	N/A	N/A	N/A	N/A	N/A

N/A not applicable

Evaluation based on the CINEAS case study in which 315 clinical symptoms were matched to Human Phenotype Ontology. The table shows the recall/precision per position in SORTA, BioPortal Annotator and ZOOMA. N.B. both BioPortal Annotator and ZOOMA have a limitation that they can only find exact matches and return a maximum of three candidates.

## Case 3: Benchmark against existing matches between ontologies

We downloaded 700 existing matches between HPO and DO concepts, 1148 matches between HPO and NCIT concepts, and 3631 matches between HPO and OMIM concepts from BioPortal. We used the matching terms from DO, NCIT and OMIM as the input values and HPO as the target coding system and generated matches using SORTA, BioPortal Annotator and ZOOMA. Supplementary Table S3 shows that all three tools managed to reproduce most of the existing ontology matches with SORTA slightly outperforming the other two by retrieving all of the ontology matches. Scrutiny revealed that SORTA was able to find the complex matches, where data values and ontology terms consist of multiple words, and some of which are concatenated, e.g. matching ‘propionic acidemia’ from DO with ‘Propionicacidemia’ from HPO. We also noticed that beyond the first rank, precision in SORTA is lower than the other two (with the highest precision in ZOOMA). In addition, we investigated what proportion of data values could be automatically matched at different cut-offs. Supplementary Table S4 shows that at similarity score cut-off of 90%, SORTA recalled at least 99.6% of the existing matches with 100% precision across all three matching experiments.

## Discussion

In RESULTS section, we have evaluated SORTA in three different use cases that demonstrated that SORTA can indeed help human experts in performing the (re)coding tasks in terms of improving the efficiency. While user evaluations of SORTA were very positive, there was still much debate among co-authors on the need to combine Lucene-based matching with n-gram post-processing and if we can make better use of ontology relationships. Below we will discuss these issues as basis for future directions to improve algorithm performance while retaining usability.

**Figure 5.**
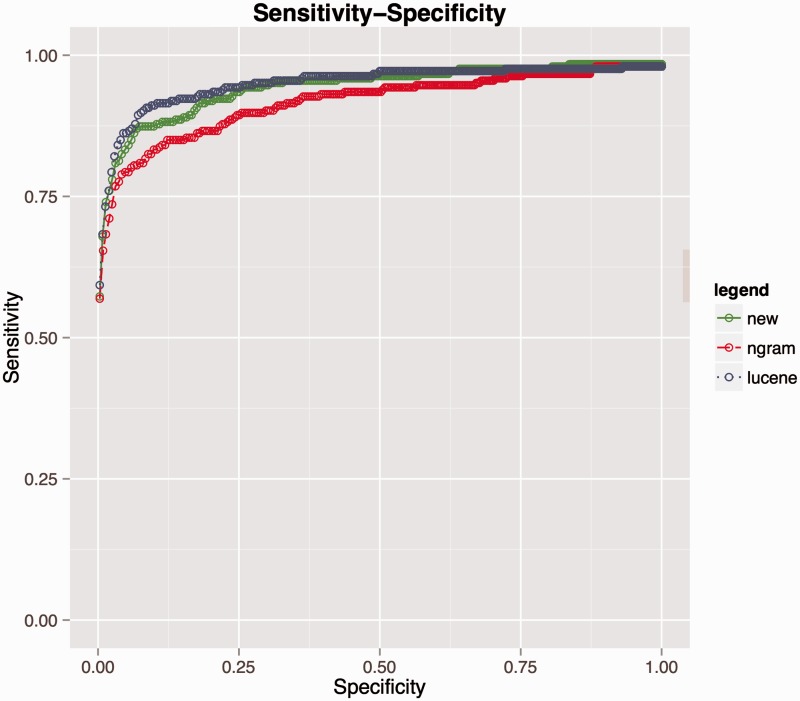
Performance comparison for matching HPO terms among three algorithms. Lucene (blue line), combination of Lucene + n-gram (red) and combination of Lucene + n-gram + inverse document frequency (green).

As mentioned in the Method section, Lucene scores were not really informative for users, but the order in which the matching results were sorted by Lucene seemed better thanks to the cosine similarity function that takes information content into account. After applying the n-gram-based algorithm, this order was sometimes changed. To evaluate this issue we performed the same matching tasks using Lucene and Lucene + n-gram. In the case of coding LifeLines data, the performances were quite similar and the inclusion of n-gram did not change the order of the matching results, see Supplementary material: PrecisionRecallLifeLines.xlsx. However, in the case of matching HPO terms, there was a large difference in precision and recall as shown in Figure 5 and Supplementary material PrecisionRecallCINEAS.xlsx. Lucene alone outperformed the combination of the two algorithms. We hypothesize that this may be caused by Lucene’s use of word inverse document frequency (IDF) metrics, which are calculated for each term (t) using the following formula:

$$\text{idf}\left(t\right)=1+\log\left(\frac{\text{total}\;{\text{Number}}_{\text{docs}}}{\text{docFreq}+1}\right)$$

where docFreq is the number of documents that contain the term.

We checked the IDFs for all the words from input values for the HPO use case and Supplementary Figure S1 shows the large difference in the information carried by each word. This suggested that, to improve the usability of the tool, we should allow users to choose which algorithm they wish to use to sort the matching results, an option that we will add in the near future. We also explored if we could simply add information content to the n-gram scoring mechanism to make the ranks consistent by redistributing the contribution of each of the query words in the n-gram score based on the IDF. For example, using n-gram the contribution of the word ‘joint’ in the query string ‘hyperextensibility hand joint’ is about 18.5% because ‘joint’ is 5/27 letters. However, if this word is semantically more important, results matching this word should have a higher score. We therefore adapted the n-gram algorithm to calculate the IDF for each of the words separately, calculate the average and reallocate the scores to the more important words as follows:

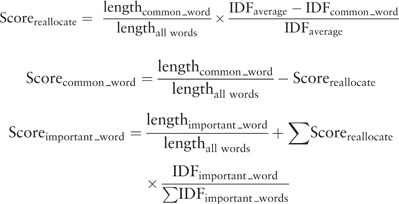

where common_word is defined as having an IDF that is lower than IDF_average _and important_words is defined as the IDF that is higher than IDF_average_.

This resulted in an improvement of recall compared to naive n-gram scoring at rank 10th from 0.79 to 0.84 (for details see Supplementary material: comparision_ngram_ lucene.xlsx), and the summarized comparison is provided via receiver operating characteristic (ROC) curve in Figure 5. However, Lucene still outperforms this metric and we speculate that this can be explained by the fundamental difference between the underlying scoring functions. The n-gram score is more sensitive to the length of input strings than Lucene and it is quite possible that two strings do not share any of the words but share similar bigram tokens, especially when dealing with long strings. Consequently, the n-gram-based algorithm might find more false positives than Lucene. However, in practice, the number of data values to be coded/recoded is quite large and the benefit of using an n-gram score cut-off value above which all the suggested matches are automatically selected outweighs this drawback.

Another issue was whether we could make better use of all the knowledge captured in ontologies. We noticed in some matching examples that related terms that come from the same ontological cluster tend to show up together in the matching results. For example, Figure 4 shows that the input term ‘external auditory canal defect’ is not matched to any of the top three candidates because they are too specific and hence we have to take the more general ontology term ‘Auditory canal abnormality’, which is actually ranked 11th, as the match even though this term is in fact the parent of the three top candidates. This indicates that if the input value is not matched by any of the candidates with a high similarity score and the candidates contain clusters of ontology terms, the parent ontology term should probably be selected as the best match (which is similar to the way human curators make decisions on such matches). However, translating this knowledge into an automatic adaptation of matching a score is non-trivial and something we plan to work on in the future.

## Conclusions

We developed SORTA as a software system to ease data cleaning and coding/recoding by automatically shortlisting standard codes for each value using lexical and ontological matching. User and performance evaluations demonstrated that SORTA provided significant speed and quality improvements compared to the earlier protocols used by biomedical researchers to harmonize their data for pooling. With increasing use, we plan to dynamically update the precision and recall metrics based on all users’ previous selections so that users can start the matching tasks with confident cut-off values. In addition, we plan to include additional resources such as WordNet for query expansion to increase the chance of finding correct matches from ontologies or coding systems. Finally, we also want to publish mappings as linked data, for example as nanopublications (26) (http://nanopub.org), so they can be easily reused. SORTA is available as a service running at http://molgenis.org/sorta. Documentation and source code can be downloaded from http://www.molgenis.org/wiki/SORTA under open source LGPLv3 license.

## Supplementary data

Supplementary data are available at *Database* online.

## Funding

This work was supported by the European Union Seventh Framework Programme (FP7/2007-2013) grant number 261433 (Biobank Standardisation and Harmonisation for Research Excellence in the European Union - BioSHaRE-EU) and grant number 284209 (BioMedBridges). It was also supported by BBMRI-NL, a research infrastructure financed by the Netherlands Organization for Scientific Research (NWO), grant number 184.021.007. We thank Anthony Brookes of Leicester University who contributed the abbreviation ‘SORTA’, and Kate Mc Intyre and Jackie Senior for editing the manuscript. Funding for open access charge: BioSHaRE-EU.

*Conflict of interest*. None declared.

## Supplementary Material

Supplementary Data
