# Supplementary material for: SORTA: a system for ontology-based re-coding and technical annotation of biomedical phenotype data
Source: Database (Oxford). 2015 Sep 17;2015:bav089. doi: 10.1093/database/bav089 (PMC4574036; doi:10.1093/database/bav089)
Supplement: Supplementary Data [file supp_2015_bav089_index.html]

SORTA: a system for ontology-based re-coding and technical annotation of biomedical phenotype data — Supplementary Data 

# SORTA: a system for ontology-based re-coding and technical annotation of biomedical phenotype data

## Supplementary Data

files

- Supplementary Data - docx file
