# Supplementary material for: SORTA: a system for ontology-based re-coding and technical annotation of biomedical phenotype data
Source: Database (Oxford). 2015 Sep 17;2015:bav089. doi: 10.1093/database/bav089 (PMC4574036; doi:10.1093/database/bav089)
Supplement: Supplementary Data [file supp_bav089_2015_7_July_SORTA_submission_version_SUPPL.docx]

# *Supplementary Material for* SORTA: a System for Ontology-based Re-coding and Technical Annotation of biomedical phenotype data by Chao Pang et al.

## Supplementary Table 1

**Precision and Recall calculated based on n-gram similarity cutoffs from 80% to 100%**

|  | Before curation | | After curation | |
| --- | --- | --- | --- | --- |
| n-gram cutoff | Recall | Precision | Recall | Precision |
| 80% | 0.22 | 0.99 | 0.78 | 0.99 |
| 81% | 0.22 | 0.99 | 0.75 | 0.99 |
| 82% | 0.22 | 1.00 | 0.73 | 0.99 |
| 83% | 0.21 | 1.00 | 0.73 | 0.99 |
| 84% | 0.20 | 1.00 | 0.71 | 1.00 |
| 85% | 0.20 | 1.00 | 0.70 | 1.00 |
| 86% | 0.19 | 1.00 | 0.68 | 1.00 |
| 87% | 0.18 | 1.00 | 0.68 | 1.00 |
| 88% | 0.18 | 1.00 | 0.67 | 1.00 |
| 89% | 0.18 | 1.00 | 0.66 | 1.00 |
| 90% | 0.17 | 1.00 | 0.65 | 1.00 |
| 91% | 0.17 | 1.00 | 0.64 | 1.00 |
| 92% | 0.17 | 1.00 | 0.63 | 1.00 |
| 93% | 0.16 | 1.00 | 0.60 | 1.00 |
| 94% | 0.16 | 1.00 | 0.58 | 1.00 |
| 95% | 0.15 | 1.00 | 0.57 | 1.00 |
| 96% | 0.14 | 1.00 | 0.56 | 1.00 |
| 97% | 0.14 | 1.00 | 0.56 | 1.00 |
| 98% | 0.14 | 1.00 | 0.56 | 1.00 |
| 99% | 0.14 | 1.00 | 0.56 | 1.00 |
| 100% | 0.14 | 1.00 | 0.56 | 1.00 |

## Supplementary Table 2

**Precision and Recall based on n-gram similarity from 88% to 100%**

| **N-gram cutoff** | **Recall** | **Precision** |
| --- | --- | --- |
| 88% | 0.33 | 0.98 |
| 89% | 0.33 | 1.00 |
| 90% | 0.32 | 1.00 |
| 91% | 0.30 | 1.00 |
| 92% | 0.29 | 1.00 |
| 93% | 0.29 | 1.00 |
| 94% | 0.29 | 1.00 |
| 95% | 0.28 | 1.00 |
| 96% | 0.28 | 1.00 |
| 97% | 0.28 | 1.00 |
| 98% | 0.28 | 1.00 |
| 99% | 0.28 | 1.00 |
| 100% | 0.28 | 1.00 |

## Supplementary Table 3

**Comparison of performances for SORTA, BioPortal Annotator and ZOOMA in recreating existing ontology matches.** The evaluation was based on three pairs of existing ontology matches: HPO/DO, HPO/NCIT and HPO/OMIM. The table shows the recall/precision per rank in SORTA, BioPortal Annotator and ZOOMA.

|  | Matching task for HPO-DO (700 matches) | | | | | | | | |
| --- | --- | --- | --- | --- | --- | --- | --- | --- | --- |
|  | SORTA | | | BioPortal Annotator | | | ZOOMA | | |
| Rank cutoff | Recall | Precision | F-measure | Recall | Precision | F-measure | Recall | Precision | F-measure |
| 1 | 0.999 | 0.999 | 0.999 | 0.964 | 0.974 | 0.969 | 0.979 | 0.994 | 0.986 |
| 2 | 1.000 | 0.500 | 0.999 | 0.964 | 0.756 | 0.847 | 0.983 | 0.984 | 0.984 |
| 3 | 1.000 | 0.330 | 0.660 | 0.964 | 0.731 | 0.832 | 0.984 | 0.983 | 0.984 |
|  | Matching task for HPO-NCIT (1148 matches) | | | | | | | | |
|  | SORTA | | | BioPortal Annotator | | | ZOOMA | | |
| Rank cutoff | Recall | Precision | F-measure | Recall | Precision | F-measure | Recall | Precision | F-measure |
| 1 | 0.997 | 0.997 | 0.997 | 0.979 | 0.988 | 0.984 | 0.987 | 0.996 | 0.992 |
| 2 | 1.000 | 0.500 | 0.667 | 0.979 | 0.792 | 0.876 | 0.988 | 0.967 | 0.977 |
| 3 | 1.000 | 0.333 | 0.500 | 0.979 | 0.770 | 0.862 | 0.989 | 0.963 | 0.976 |
|  | Matching task for HPO-OMIM (3631 matches) | | | | | | | | |
|  | SORTA | | | BioPortal Annotator | | | ZOOMA | | |
| Rank cutoff | Recall | Precision | F-measure | Recall | Precision | F-measure | Recall | Precision | F-measure |
| 1 | 0.996 | 0.996 | 0.996 | 0.670 | 0.974 | 0.794 | 0.976 | 0.993 | 0.984 |
| 2 | 1.000 | 0.500 | 0.667 | 0.670 | 0.761 | 0.713 | 0.980 | 0.987 | 0.983 |
| 3 | 1.000 | 0.333 | 0.500 | 0.670 | 0.732 | 0.700 | 0.980 | 0.986 | 0.983 |

*HPO Human Phenotype Ontology;*

*DO : Disease Ontology;*

*NCIT : National Cancer Institute Thesaurus;*

*OMIM : Online Mendelian Inheritance in Man*

## Supplementary Table 4

**Evaluation of performance for SORTA at different percentage cutoff values**

| Cut-off percentage | HPO-DO  (700 matches) | | HPO-NCIT  (1148 matches) | | HPO-OMIM  (3631 matches) | |
| --- | --- | --- | --- | --- | --- | --- |
|  | Recall | Precision | Recall | Precision | Recall | Precision |
| 100% | 0.990 | 1.000 | 0.993 | 1.000 | 0.995 | 1.000 |
| 90% | 0.996 | 1.000 | 0.999 | 1.000 | 0.996 | 1.000 |
| 80% | 0.999 | 0.999 | 1.000 | 1.000 | 1.000 | 1.000 |

*HPO Human Phenotype Ontology;*

*DO : Disease Ontology;*

*NCIT : National Cancer Institute Thesaurus;*

*OMIM : Online Mendelian Inheritance in Man*

## Supplementary Figure 1

**The inverse document frequency (IDF) for the input query words.** The IDF is first calculated for all the words available from Human Phenotype Ontology (HPO) to create the IDF library, then all of the words from the input query are checked against this library to create the plot.

## Supplementary material external files

Six Microsoft Excel files LifeLines_MET_mappings.xlsx, PrecisionRecallLifeLines.xlsx, PrecisionRecallCINEAS.xlsx, CINEAS_HPO_Rank_summary.xlsx

and

CINEAS_HPO_Manual_mappings.xlsx, comparision_ngram_lucene.xlsx,

comparision_ngram_lucene.xlsx

and

one zip file ontology_mapping.zip can be found at <https://molgenis26.target.rug.nl/downloads/sorta/supplementary_material>
